# Supplementary figures and images for: Evaluation of 8-Channel Radiative Antenna Arrays for Human Head Imaging at 10.5 Tesla
Source: Sensors (Basel). 2021 Sep 8;21(18):6000. doi: 10.3390/s21186000 (PMC8469352; doi:10.3390/s21186000)

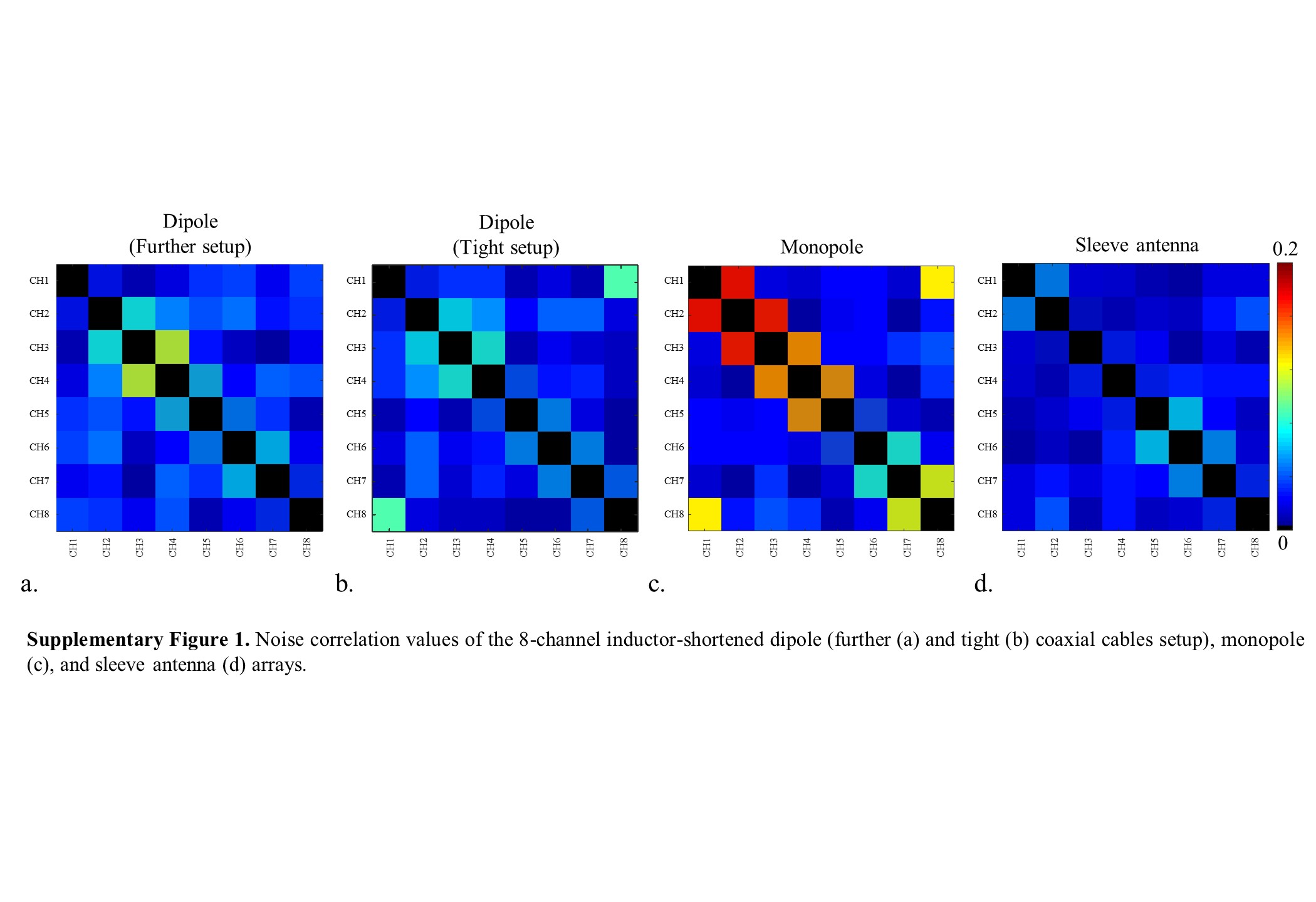

Supplement: Supplementary file 1 [file sensors-21-06000-s001.zip › S. Fig.1.jpg]

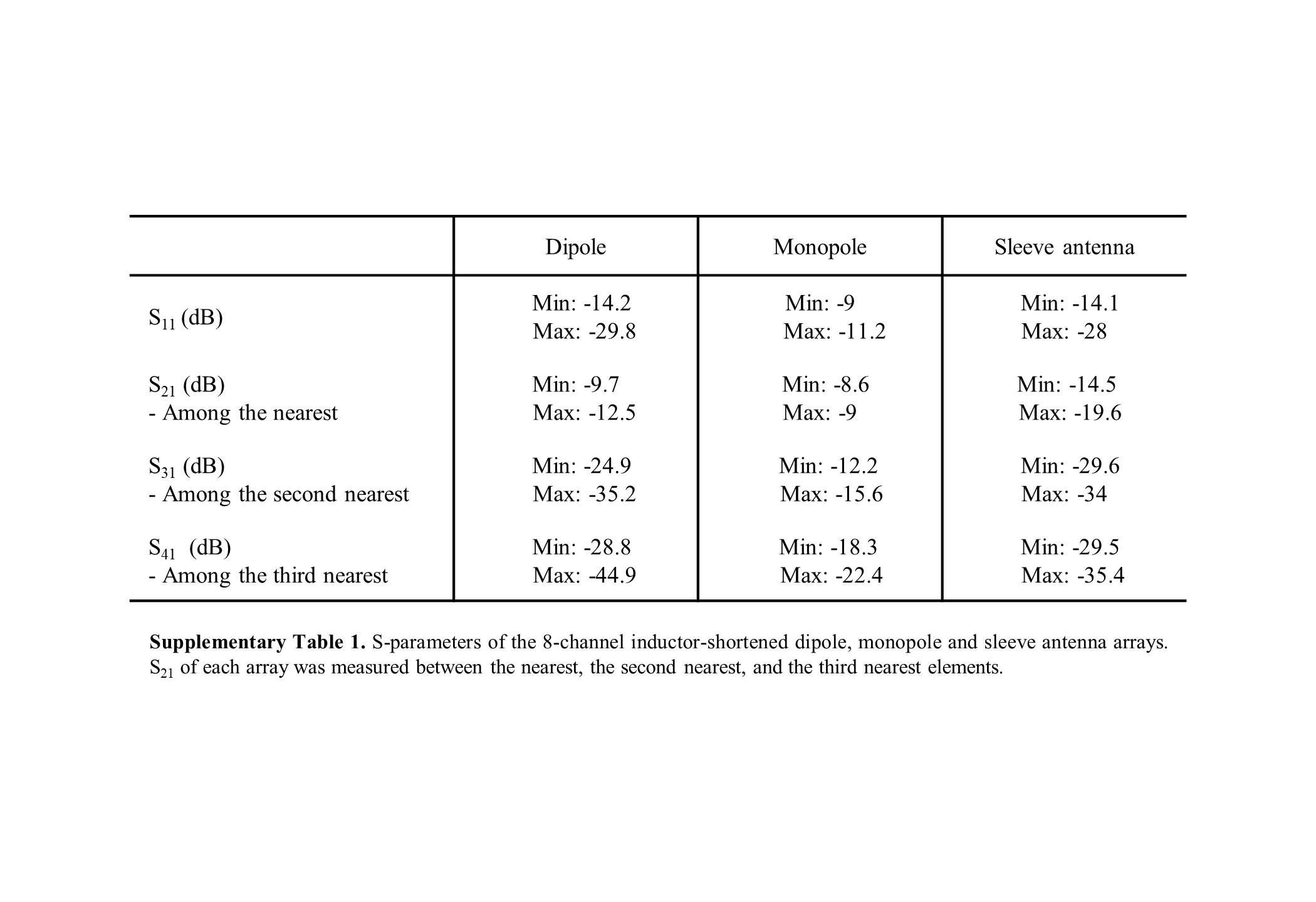

Supplement: Supplementary file 1 [file sensors-21-06000-s001.zip › S. Table1.jpg]
